# Implications of ABCC4–Mediated cAMP Efflux for CRC Migration

**DOI:** 10.3390/cancers12123547

**Published:** 2020-11-27

**Authors:** Jakub Kryczka, Ewelina Sochacka, Izabela Papiewska-Pająk, Joanna Boncela

**Affiliations:** 1 Institute of Medical Biology, Polish Academy of Sciences, 93-232 Lodz, Poland; jkryczka@cbm.pan.pl (J.K.); esochacka@cbm.pan.pl (E.S.); ipapiewska-pajak@cbm.pan.pl (I.P.-P.); 2 Faculty of Biology and Environmental Protection, University of Lodz, 90-236 Lodz, Poland

**Keywords:** ABC transporters, ABCC4 protein, colon cancer, metastasis

## Abstract

**Simple Summary:**

Cancer cells have developed a number of mechanisms to overcome anticancer therapy; the active efflux of drugs from cells via multidrug resistance proteins (MRPs) is one of them. MRPs belong to the superfamily of ATP binding cassette (ABC) proteins. It was hypothesized that the inhibition of ABC drug transporter activity during cancer therapy could sensitize drug-resistant tumors and/or improve the initial activity of anticancer agents. We demonstrated that the pharmacological inhibition of ABCC4 increases the migratory rate and invasive protrusion formation in colorectal cancer (CRC). Thus, during the use of ABCC4 inhibitors to reduce chemotherapy resistance or drugs that are potential substrates of ABCC4, the indirect effect on cancer metastasis should be taken into consideration and may be important in selecting a therapy scheme for patients.

**Abstract:**

Colorectal cancer (CRC) presents significant molecular heterogeneity. The cellular plasticity of epithelial to mesenchymal transition (EMT) is one of the key factors responsible for the heterogeneous nature of metastatic CRC. EMT is an important regulator of ATP binding cassette (ABC) protein expression; these proteins are the active transporters of a broad range of endogenous compounds and anticancer drugs. In our previous studies, we performed a transcriptomic and functional analysis of CRC in the early stages of metastasis induced by the overexpression of Snail, the transcription factor involved in EMT initiation. Interestingly, we found a correlation between the Snail expression and ABCC4 (MRP4) protein upregulation. The relationship between epithelial transition and ABCC4 expression and function in CRC has not been previously defined. In the current study, we propose that the ABCC4 expression changes during EMT and may be differentially regulated in various subpopulations of CRC. We confirmed that ABCC4 upregulation is correlated with the phenotype conversion process in CRC. The analysis of Gene Expression Omnibus (GEO) sets showed that the *ABCC4* expression was elevated in CRC patients. The results of a functional study demonstrated that, in CRC, ABCC4 can regulate cell migration in a cyclic nucleotide-dependent manner.

## 1. Introduction

Colorectal cancer (CRC) treatment is challenging due to the heterogeneous nature of cancer, in which prognosis depends on the tumor type and disease stage. Despite progress in diagnosis and therapy, metastasis and chemoresistance are two critical processes for the overall survival of CRC patients [1]. Approximately 50–60% of patients who are diagnosed with CRC will eventually develop metastatic disease. Most often, metastases develop after first-line chemotherapeutic drug and monoclonal antibody treatments for local disease. Over several years, many studies have demonstrated that metastatic CRC presents significant molecular heterogeneity [2,3]. This observation explains the enormous variability noted in regard to treatment outcomes. The cellular plasticity of epithelial to mesenchymal transition (EMT) is one of the key factors responsible for the heterogeneous nature of the metastatic CRC phenotype. EMT is not a binary process [4]. The epithelial cells undergoing EMT give rise to cell populations that may enter into states with various proportions of epithelial and mesenchymal features. Metastatic cells represent various EMT states, from epithelial-like through to mixed epithelial/mesenchymal (hybrid) to a strongly mesenchymal phenotype. Hybrid and mesenchymal cells exhibit increased migratory and invasive features, suggesting a detrimental role of EMT during metastatic dissemination [5,6]. Another complicating factor is that EMT has been linked to additional traits that are not associated with canonical EMT regulation, such as stemness and resistance to anticancer therapeutic drugs [7].

Cancer cells have developed a number of mechanisms to overcome anticancer therapy, and the active efflux of drugs from cells via multidrug resistance proteins (MRPs) is one of them. MRPs belong to the superfamily of ATP-binding cassette (ABC) proteins, active transporters with a broad range of substrate spectra, including anticancer drugs [8]. The human genome contains 48 ABC genes, and they are classified into seven subfamilies (ABCA-ABCG) [9]. Among them, ABCB1, ABCC1, and ABCG2 are highly involved in the acquisition of multidrug resistance (MDR). Increased ABC transporter expression has been correlated with aggressive and invasive cancers. EMT is an important regulator of ABC transporters, and the expression of ABC transporters changes continuously during EMT [10,11]. Mechanistically, it was demonstrated that the promoters of ABC transporters carry several binding sites for EMT-inducing transcription factors, and the overexpression of Twist, Snail, and ZEB increases the promoter activity of ABC transporters [12,13]. In our previous studies, we performed transcriptomic and functional analyses of CRC in the early stages of metastasis induced by the overexpression of Snail, the transcription factor involved in EMT initiation. Our results showed that Snail regulates early phenotype conversion towards a hybrid EMT. Interestingly, we found a correlation between Snail expression and ABCC4 (MRP4) protein upregulation [14]. The relationship between epithelial transition and ABCC4 expression and function in CRC has not been previously defined. In the current study, we propose that ABCC4 expression changes during EMT and may be differentially regulated in various subpopulations of CRC. ABCC4 is able to transport a range of organic anionic compounds out of the cell; thus, most functional studies of ABCC4 have classically focused on its role in cancer chemotherapy [15]. However, the physiological actions of this protein are quite diverse, and drug transport appears not to be the most important evolutionarily conserved function. The efflux of cyclic adenosine monophosphate (cAMP) through ABCC4 has been well documented in various cell types, suggesting that this transporter plays a relevant role in the regulation of cAMP signaling. ABCC4 was shown to modulate the compartmentalization of cAMP signaling in a colon adenocarcinoma cell line (HT29, T84), and ABCC4 inhibition with MK571 compound leads to the accumulation of cAMP at or near the plasma membrane [16]. Notably, the inhibition of ABCC4 function through MK571 or gene knockout was shown to have a direct role in cell migration. The pharmacological inhibition of ABCC4 with MK571 resulted in the intracellular accumulation of cAMP, leading to increased fibroblast migration related to protein kinase A (PKA) activity [17,18]. In the current paper, we show that in CRC, similar to what has been observed in fibroblasts, ABCC4 can regulate cell migration in a cAMP-dependent manner.

The understanding of the mechanistic linkage between two phenomena, ABCC4 transport function and cell migration, would significantly contribute to the improvement of anticancer therapy in CRC. Numerous ABC transporter inhibitors have been developed and tested [19]. The clinical use of ABC transporter inhibitors is still an ongoing challenge, partially due to the intratumor heterogeneity; thus, the evaluation of ABCC4 expression status alone or in combination with other transporters in various CRC subpopulations supported by information on signaling pathways related to ABCC4 transport function may improve the development of patient-tailored therapy.

## 2. Results

### 2.1. ABCC4 Is Overexpressed in CRC

**Figure 1:**
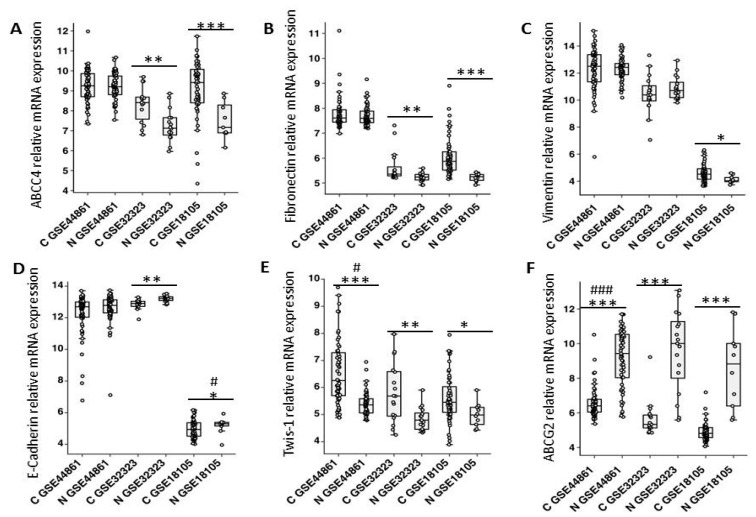
ABCC4 (**A**), ABCG2 (**F**), and EMT marker (**B**–**E**) mRNA expression in CRC and normal tissue. Microarray data from the public Gene Expression Omnibus (GEO) database (GSE18105, GSE44861, and GSE32323: [20] were analyzed with the respective *n* for c (cancer) and n (normal): GSE18105 n_c_ = 57 n_n_ = 10 (primary tumors only); GSE44861 n_c_ = 56, n_n_ = 55; and GSE32323 n_c_ = 17 n_n_ = 17. Normality test (Shapiro–Wilk) was performed, followed by the Mann–Whitney U test (*)—* *p* < 0.05; ** *p* < 0.005; *** *p* < 0.001, no statistically significant—no indicator. Additionally, all the normally distributed samples were tested using *t*-test (#) # *p* < 0.05; ### *p* < 0.001. Data density distribution is presented in the Supplementary Materials, Figure S2.

To identify the expression level of *ABCC4* in CRC, we first analyzed the *ABCC4* expression levels in normal and CRC tissues by a bioinformatics analysis. Microarray data from the public Gene Expression Omnibus (GEO) database (GSE18105, GSE44861, and GSE32323: [20] revealed that *ABCC4* was significantly upregulated in primary tumors compared to normal tissues (Figure 1A). Since we previously observed that ABCC4 expression was upregulated in HT-29 colon cancer cells stably overexpressing Snail and that those cells had transcriptomic profile changes indicating EMT induction [14], we examined the same GEO database to identify mRNA related to EMT, whose expression was changed in our cell line model. Given the previous results, we found that the mRNA levels of the mesenchymal markers fibronectin (Figure 1B) and vimentin (Figure 1C) were elevated and that the mRNA levels of E-cadherin (epithelial marker, Figure 1D) were decreased in the analyzed CRC tissue compared to normal tissue. Among the transcription factors involved in EMT, we found an increase in the expression of *Twist* mRNA in the analyzed CRC patient samples (Figure 1E). Thus, we confirmed that the elevated expression of *ABCC4* in the analyzed CRC data sets was related to changes in phenotypic transition markers. EMT is induced by different stimuli, and TGFβ is its canonical driver. In our previous study, TGFβ was indicated by ingenuity pathway analysis as the most significant upstream regulator of the transcriptomic changes in response to Snail in HT29 cells, and the changes in the expression of the TGFβ signaling pathway components indicated that this pathway was modestly activated [14].

**Figure 2:**
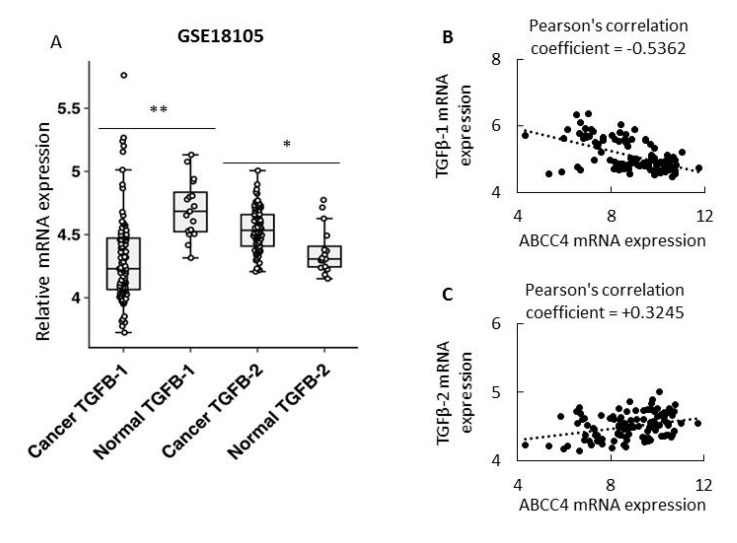

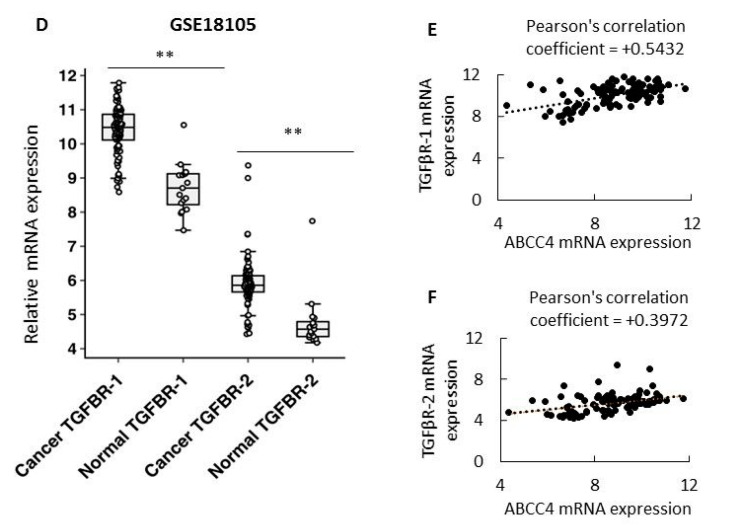
TGFβ pathway and ABCC4 mRNA expression analysis. TGFβ1/2 (**A**) and TGFβR1/2 (**D**) expression analysis in CRC patient tissue. Data obtained from the GSE18105 data set, cancer *n* = 94, normal *n* = 16. Correlation of ABCC4 expression and TGFβ1 (**B**) or TGFβ2 (**C**) or TGFβR1 (**E**) or TGFβR2 (**F**) expression, *n* = 110 [20]. Sample data sets were tested with the Shapiro–Wilk test, presenting a normal distribution, followed by the *T*-test. Pearson’s correlation coefficient (PCC): 0–0.25 no PCC, 0.25–0.5 low PCC, 0.5–0.75 moderate PCC, 0.75-1 strong PCC. Comparison of Pearson’s and Spearman’s correlation values in Figure S1A, Supplementary Materials. Data density distribution (Figure 2A,D) produced with SinaPlot is presented in Supplementary Materials, Figure S2. * *p* < 0.05; ** *p* < 0.005.

To check whether the *ABCC4* expression upregulation is related to the TGFβ signaling pathway in CRC, we analyzed GSE18105 datasets—which represent the statistically most significant differences in EMT markers between normal and cancer cells—for the expression of TGFβ1/2 and their receptors. We observed a significant upregulation of *TGFβ2* and both TGFβ receptors (*TGFβR1* and *TGFβR2*) in tumors compared to normal colon tissue (Figure 2A,D). Furthermore, we noticed a positive correlation between the *ABCC4* expression and *TGFβ2* and both the TGFβ receptors’ (*TGFβR1/2*) expression (Figure 2C–F and Figure S1A), and a negative correlation with the *TGFβ1* expression (Figure 2B and Figure S1A). These results confirmed that *ABCC4* expression is related to the TGFβ-induced transcriptomic signature in CRC. To date, in clinical studies ABCG2 has been recognized as the main drug efflux protein in CRC [21]. However, studies comparing the expression of *ABCG2* mRNA in normal colon tissue and tumor tissue showed that primary colon cancer cells exhibit an initial downregulation of *ABCG2* mRNA expression [22]. Our previous results showed that HT29 lines with upregulated Snail expression exhibited an increase in *ABCC4* expression and a decrease in *ABCG2* expression [14]. To validate this observation, we analyzed the same datasets from the GEO database (GSE18105, GSE448, and GSE32323) and found that *ABCC4* was significantly upregulated while *ABCG2* was downregulated in primary tumors compared to normal colon tissues. [20] A representative analysis is presented in Figure 1F. These data may indicate that ABCC4 is a prevalent drug transporter in primary tumors in which ABCG2 is downregulated, and confirm the hypothesis that the changes in the ABC transporter expression might be related to the various stages of CRC progression. Next, to correlate the transcriptomic analysis to the ABCC4 protein function in CRC, we analyzed the ABCC4 protein expression profile and performed a functional study.

### 2.2. ABCC4 Protein Expression in CRC Is Related to Phenotypic Transition

**Figure 3:**
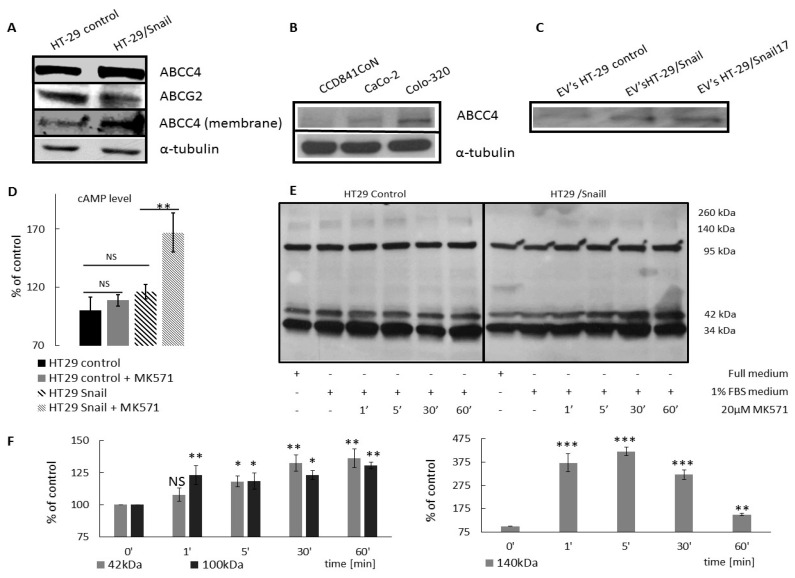
ABCC4 protein expression level in CRC cell lines. Western blot performed in standard reducing SDS PAGE conditions using goat anti ABCC4 (#PA5 18315, Thermo Scientific) and rabbit anti ABCG2 (#ORB 155559 Biorbyt). (**A**) Protein expression level of ABCC4 and ABCG2 in HT-29 stably overexpressing transcription factor Snail (HT29/Snail) and control HT-29. ABCC4 level in the membrane fraction (obtained by biotynylation using EZ-Link Sulfo -NHS-Biotin Thermo Scientific kit) of HT-29 control cells and HT-29 Snail *n* = 3. (**B**) ABCC4 protein expression level in CRC cells in different states of EMT: CCD841CoN (most epithelial), CaCo-2 (moderate EMT), and Colo-320 (most mesenchymal) *n* = 3. (**C**) ABCC4 protein abundance in Extracellular Vesicles (EVs) released from HT-29 control cells and two HT-29 stably overexpressing transcription factor Snail clones (HT-29/Snail and HT-29/Snail17), *n* = 2. (**D**) Intracellular cAMP level measurement. Accumulation of cAMP in HT29 cells was measured using a cAMP competitive kit (#581001 Cayman Chemical). Cells were incubated for 24 h with MK571 20 µM, or untreated ones were assayed according to the manufacturer’s protocol. Calculation were conducted using the Cayman data sheet. cAMP concentration of HT29 was set as 100%. *T*-test performed, *n* = 5; * *p* < 0.05; ** *p* < 0.005; *** *p* < 0.001. NS—not statistically significant. (**E**) PKA phosphorylation profile analysis. HT29 Snail cells were seeded on a 6-well plate. Then, 24 h after, full growth medium was changed into starving (FBS free) medium for 24 h. Next, 20uM of MK571 was added to cells for 60, 30, 5, and 1 min. Cells without the starving procedure were used as a positive control, and negative control cells were not treated with MK571. Phosphorylation profile analysis was performed using phospho-(ser/thr) PKA Substrate Antibody #9621 (Cell Signaling Technology). Significant time- (exposure) related impact on the phosphorylation profile was observed for 42 kDa and 95–100 kDa proteins in HT29 Snail cells compared to no time-related changes in control cells, *n* = 3. (**F**) HT-29/Snail PKA phosphorylation profile analyzed with densitometry; statistical significance estimated using *T*-test. * *p* < 0.05; ** *p* < 0.005; *** *p* < 0.001. NS—not statistically significant.

The ABCC4 protein was identified as an active transporter of cyclic nucleotides and as a mediator of secondary messenger signaling through cAMP in several different cell and tissue types [13]. To determine this function of ABCC4 in CRC, we first analyzed the level of ABCC4 protein in HT29 cells. We observed an increased ABCC4 protein expression in HT29 cells overexpressing Snail (HT29/Snail) compared to control HT29 cells (Figure 3A, Figure S1B, Supplementary Materials). Next, as our previous mRNA analysis of HT29/Snail cells [14] and current bioinformatics analysis of CRC patient samples showed that the upregulation of *ABCC4* accompanied the downregulation of *ABCG2*, we also performed a Western blot analysis of the ABCG2 protein. We observed that this protein was present at lower levels in HT29/Snail cells than in control HT29 cells (Figure 3A and Figure S1B, Supplementary Materials). In our previous study, we found a correlation between Snail expression and the upregulation of *ABCC4* [14]. However, crucial EMT-activating transcription factors, including Snails, ZEB, and Twist, recognize the E-box DNA sequences in the promoter region of the *ABCC4* gene, and the bioinformatic analysis of the *ABCC4* promoter region revealed the presence of 11 E-box sequences [12]; thus, we assume that the *ABCC4* upregulation is correlated with the epithelial reprogramming process rather than with the activity of a single transcription factor during epithelial transition in CRC. To check this hypothesis, we performed an ABCC4 protein expression analysis in various CRC cell lines representing epithelial, intermediate mesenchymal, and strongly mesenchymal phenotypes. All of these cell lines are directly derived from primary colorectal cancers of different clinical stages and differentiation grades [23,24]. Our results showed that cells with an epithelial phenotype (CCD841CoN) expressed less ABCC4 than intermediate or strongly mesenchymal cells (CaCo-2 and Colo-320, respectively) (Figure 3B and Figure S1C, Supplementary Materials), suggesting that the ABCC4 expression is related to the phenotypic status in CRC. The highest expression level was noted for Colo-320 cells. Interestingly, Colo-320 was shown to have the strongest expression of the EMT signature and the highest propensity to local invasion among the analyzed group of cells [25].

### 2.3. Cellular Localization of ABCC4 in CRC

The cellular localization of ABCC4 regulates cAMP signaling involved in cell migration [18]. Thus, we analyzed the ABCC4 cellular localization in CRC by isolating subcellular fractions and measuring the level of ABCC4 protein, particularly in the outer membrane fraction, in comparison to whole cell extracts (input). We performed cell surface protein biotinylation using EZ-Link Sulfo-NHS-Biotin, and we collected the biotinylated protein fraction with streptavidin agarose. Using Western blot analysis, we detected higher levels of ABCC4 protein in the membrane fraction of HT29 cells overexpressing Snail than in that of the HT29 control cells (Figure 3, Figure S1B, Supplementary Materials). These results indicate that the higher expression of ABCC4 protein determines its membranous localization and transport function in cells that acquire mesenchymal traits.

Generally, the elevated expression of ABC is attributed to drug resistance. Recent data have shown that ABC protein enrichment was present in EVs from drug-resistant cells [26]. We estimated the ABCC4 abundance in extracellular vesicles (EVs) released from HT29 cells. We detected ABCC4 in HT29-derived EVs (Figure 3C, Figure S1D, Supplementary Materials). These EVs were positive for CD63, CD9, and CD81 and negative for cytochrome c and were used in our previous study for mRNA and miRNA analysis [14,27]. Our results showed a higher level of ABCC4 in EVs from two clones of HT29 cells stably overexpressing Snail, suggesting that ABCC4 is packed into CRC EVs and that the level of ABCC4 in EVs correlates with the level of ABCC4 expression in the cells.

### 2.4. Analysis of Intracellular Accumulation of cAMP

The inhibition of ABCC4 function by MK571, a known ABCC4 inhibitor, has been used in previous works and was shown to increase the intracellular cyclic nucleotide level and have a direct role in mouse fibroblast migration. The effect was more profound, however, on the cAMP level than on the cGMP level, indicating a higher affinity of ABCC4 for cAMP. In our experiments, we noted that treatment with MK571 increased the intracellular level of cAMP in HT29 cells. These results demonstrated that ABCC4 was responsible for cAMP efflux in CRC. However, the effect was more pronounced in HT29 cells that acquired mesenchymal characteristics by Snail overexpression than in control HT29 cells (Figure 3D). We calculated the intracellular level of cAMP after 24 h of incubation with MK571 and used the cAMP competitive test (Cyclic AMP ELISA Kit, #581001, Cayman Chemicals). The concentrations of MK571 used were selected from previous reports and did not affect the cellular viability (data not shown) [28].

### 2.5. ABCC4 Function Is Necessary for Adequate PKA Activity

Intracellular cAMP has a vast repertoire of effectors; among them, the PKA enzyme family is one of the most studied, and its activity is directly related to serine/tyrosine phosphorylation [29]. Previous reports described a correlation between PKA activity and fibroblast migration [18]. Therefore, we studied whether inhibition of ABCC4 and cAMP efflux might modulate the PKA activity. HT29 cells overexpressing Snail were incubated in the presence or absence of MK571 (20 μM for 0, 1, 5, 30, and 60 min). Next, phosphorylated substrates of PKA (pPKA-Subs) were visualized using Western blot and phospho-(ser/thr) PKA Substrate Antibody (Cell Signaling) (Figure 3e, Figure S1E). The obtained results showed that in HT29 cells with Snail overexpression, short incubation (1 and 5 min) with MK571 resulted in an apparent increase in the phosphorylation of 140 kDa proteins. This effect was further decreased after 30 and 60 min. Additionally, we observed a gradual increase in the phosphorylation of 42 kDa and 140 kDa proteins, Figure 3F. Changes in the phosphorylation of PKA substrates after ABCC4 inhibition were not observed in control HT29 cells (Figure 3E, Figure S1E). This observation suggests that ABCC4 activity is necessary for the early regulation of PKA activity in cells that acquire a mesenchymal phenotype and indicates that in CRC, similar to what was observed in fibroblasts, the inhibition of ABCC4 may increase cell migration.

### 2.6. Analysis of the Migratory Potential of CRC Subtypes Treated with ABCC4 Inhibitor

**Figure 4:**
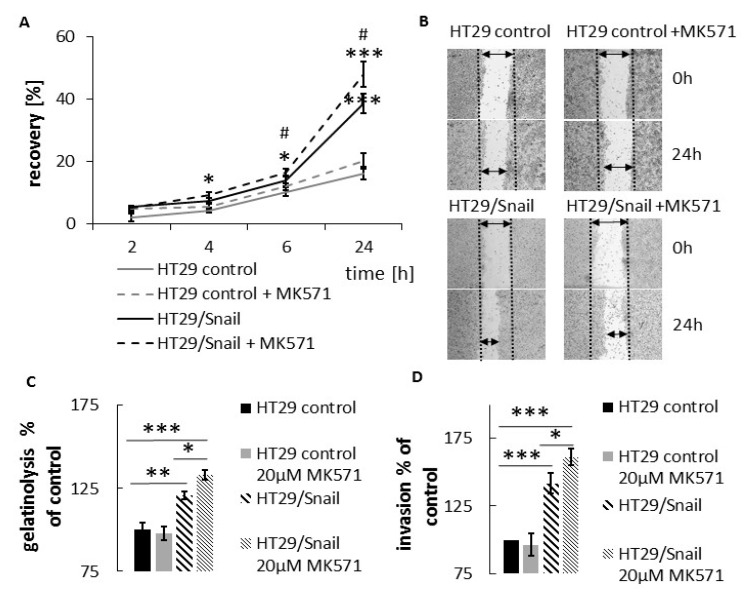

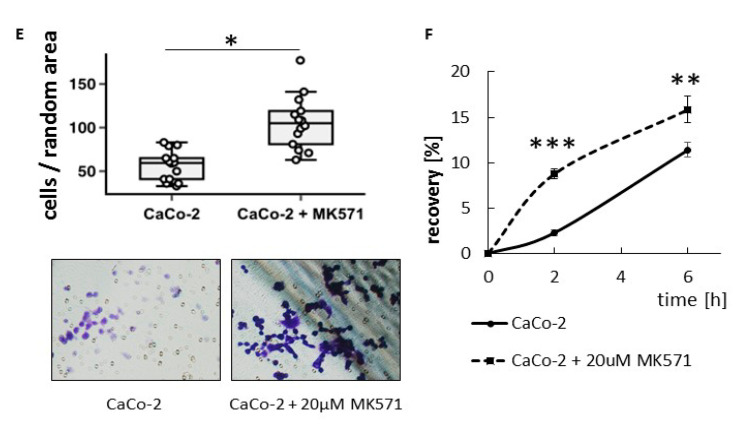
MK571 impact on CRC cells migratory abilities. (**A**) HT29 cells (control or overexpressing Snail) were grown to confluence on 6 well plate, next, wounded across the cell monolayer. New medium containing 20 μM MK571 was added. Wounded area was visualized after 0, 2, 4, 6 and 24 h—and presented in using Nikon Eclipse TE 2000-U microscope (Nikon, Japan) and calculated by ImageJ software [32]. Cell motility was estimated through the quantification of the % of recovery using the equation: R (%) = [1 − (wound area at Tt/wound area at T0)] × 100,where T0 is the wounded area at 0 h and Tt is the wounded area after t; *n* = 3; * HT-29/Snail (w/wo MK571) vs. HT-29 control; # HT-29/Snail MK571 vs. HT-29/Snail * *p* < 0.05; ** *p* < 0.005; *** *p* < 0.001, NS—no statistically significant. (**B**) Representative picture of wound healing assay. (**C**) Gelatinolysis mediated by HT-29 measured by in-situ zymography. The pericellular proteolytic abilities of HT-29 were analyzed by a measure of the increase in FITC fluorescent intensity from digested DQ gelatine relativized to control cells presented as 100%; *n* = 3 (**D**) HT29 cells, transwell assay. Cells incubated for 24 h with MK571 20 µM, or untreated once were seeded on Matrigel coated transwell inserts in the upper chamber in medium supplemented with 0.1% bovine albumin serum (BSA) (w/wo 20 µM MK571). Full medium in lower chamber served as chemoattractant for cell invasion. Membrane were cut out and all cells from membrane were calculated after 6 h of incubation followed by hematoxylin/eosin staining. Number of control HT-29 cells that transmigrate into transwell membrane through 8 µM pores covered with Matrigel was set as 100%, next number of other cells was calculated and presented as % of control. (**E**) CaCo-2 cells, transwell assay. Cells were incubated for 24 h with MK571 20 µM, or untreated once were seeded on un-coated transwell inserts (8 µM pores) in the upper chamber in 2% BSA medium (w/wo 20 µM MK571). Full medium in lower chamber served as chemoattractant for cell migration. Cells were calculated in randomly assigned areas after 3 h of incubation followed by hematoxylin/eosin staining. Interquartile range (Q1–Q3) is shown as gray box with median (Q2) with all data points from all (*n* = 3) experiments overlap on the box plot. (**F**) CaCo-2 cells, wound healing assay. Cells were seeded on collagen coated 6-well plates to full confluence. Next wound was done across cell monolayer, rinsed with phosphate buffer (PBS) PBS. Next fresh medium w/wo 20 µM MK571 was added. Cells were visualized every 2 h and % of wound enclosure was calculated as in A). * *p* < 0.05; ** *p* < 0.005; *** *p* < 0.001, NS—not statistically significant.

Given the obtained results, we further evaluated the ability of the cells to migrate in the presence of MK571. First, we performed a scratch (wound healing-like) assay. One of the major advantages of this simple method is that it mimics the migration of cells in vivo [30]. We also decided to test the impact of MK571 on the ability to cross anatomical boundaries using a gelatinolysis assay and transwell invasion assay, performed as described in our previous papers [14,28,31]. The obtained results indicated that MK571 increased the motility of both HT29 and HT29 cells overexpressing Snail (Figure 4A,B). However, the effect of inhibition on cell invasiveness and gelatinolysis activity was detected only in HT29-Snail cells (Figure 4C,D), suggesting that cells with acquired mesenchymal characteristics are more prone to ABCC4 inhibition than cells with epithelial characteristics. To confirm this observation, we investigated the motility of Caco2 cells, which represent an intermediate mesenchymal phenotype [23,24]. We observed that MK571 also increased CaCo2 migration, as detected in the transwell migration assay (Figure 4E). Since the wound healing assay is not recommended for this cell line due to its growth characteristics, we performed this assay using collagen type I-coated 6-well plates (Corning) that increased cell adhesion, preventing spontaneous detachment. MK571-treated CaCo2 cells presented a higher migration rate, as measured by faster wound closure than control cells (Figure 4F).

**Figure 5:**
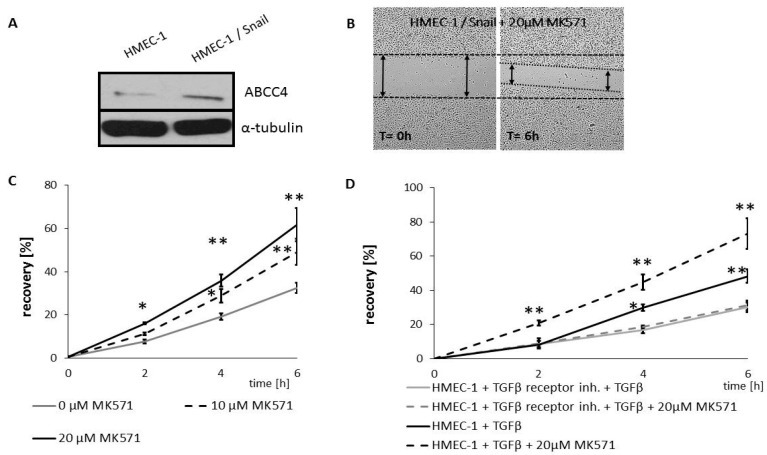
MK571 increased motility of EndoMT undergoing HMEC-1 cells. ABCC4 level in HMEC-1 and HMEC-1 overexpressing Snail cells (HMEC-1/Snail). (**A**) HMEC-1 were grown to confluence on 6-well plate, and transiently transfected with pcDNA/Snail and wounded across monolayer as described in [31]. New medium containing MK571 was added. (**B**) Representative image of HMEC-1 control or HMEC-1/Snail cells in wound healing assay. (**C**) Wounded area was visualized after 0, 2, 4 and 6 h using Nikon Eclipse TE 2000-U microscope (Nikon, Japan) and calculated by ImageJ software [32]. Cell motility was estimated through the quantification of the % of recovery using the equation: R(%) = [1 − (wound area at Tt/wound area at T0)] × 100,where T0 is the wounded area at 0 h and Tt is the wounded area after 2 or 4 h. * *p* < 0.05; ** *p* < 0.005; *n* = 3. (**D**) HMEC-1 treated w/wo TGF-β receptor inhibitor were grown to confluence on 6 well plate, incubated for 48 h with 10ng/mL TGF-β2 in starving condition as described in [31 and wounded. Wounded area was visualized and analyzed as in (**C**).

To extend this analysis, we more comprehensively evaluated the extent that ABCC4 expression and function correlated with phenotypic transition. We examined the effect of MK571 on endothelial cells upon endothelial to mesenchymal transition (EndoMT). For this purpose, we used HMEC-1 (microvascular endothelial) cells shifted towards the mesenchymal phenotype, which was broadly characterized in our previous study [31]. We investigated the migratory ability of HMEC-1 cells with transient Snail overexpression (Figure 5A–C) or TGFβ stimulation (Figure 5D) in the presence or absence of MK571. To omit any noncanonical impact of TGFβ on EndoMT, HMEC-1 cells treated with TGFβ receptor inhibitor 24 h (SB431542 #1614, Tocris Bioscience, Bristol, UK)) prior to the experiment were used as a control for the TGFβ-mediated EndoMT (as in our previous study [31]). The results showed that ABCC4 inhibition increased the migration of endothelial cells that acquire a mesenchymal phenotype.

### 2.7. Irinotecan Treatment and CRC Migration

**Figure 6:**
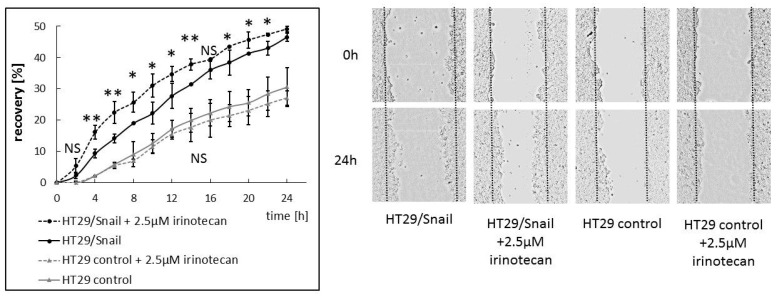
Irinotecan affects CRC migration. HT29 control and HT29/Snail cells were seeded on 24 well plate to confluence for 24 h. Next, wound was done across monolayer and fresh medium was added w/wo 2.5 µM irinotecan (final concentration). Wounded area was visualized after every 2 h by Spark multimode microplate reader (TECAN, Swizerland). Wounded area was calculated by ImageJ software [32]. Cell motility was estimated through the quantification of the % of recovery using the equation: R(%) = [1 − (wound area at Tt/wound area at T0)] × 100,where T0 is the wounded area at 0 h and Tt is the wounded area after 2 or 4 h. * *p* < 0.05; ** *p* < 0.005, NS—no statistically significant (all HT29 control vs HT29 control + 2.5 µM irinotecan were considered NS), *n* = 3.

Interestingly, irinotecan, a chemotherapeutic drug for CRC, has a high affinity for ABCC4 and was demonstrated (through substrate competition with cAMP) to increase cAMP levels at or near the plasma membrane to levels comparable with the effect of the ABCC family inhibitor MK571 [33]. Irinotecan does not directly inhibit ABCC4 transport and we assume that the endpoint effect of cAMP-mediated signaling may be similar. To test this hypothesis, we investigated whether irinotecan affected the migration of HT29 cells in a manner comparable to that of MK571. First, we established irinotecan cytotoxicity (IC25 and IC50) for HT29/Snail and control HT29 cells (Figure S1E, Supplementary Materials) using a WST-1 assay. The obtained results indicated that both HT29 variants presented similar levels of irinotecan tolerance with IC50 values of approximately 5.5 µM, which corresponds to the literature data [34]. Finally, we tested the impact of irinotecan on migration. Control HT29 cells and HT29 cells overexpressing Snail were seeded on 24-well plates for 24 h to reach confluence. Next, wounds were made across monolayers, the cells were washed with PBS and fresh medium was added w/wo 2.5 µM irinotecan (Figure 6). We decided to use a concentration of 2.5 µM, corresponding to the IC25, to avoid increased cytotoxic/cytostatic effects in the scratch assay. We noticed that irinotecan enhanced migration of HT29 overexpressing Snail, whereas the migration of control HT29 was not significantly changed. Of note, the most statistically significant increase was observed within the first 8 h of irinotecan supplementation.

## 3. Discussion

During the multistep progression of carcinomas that are initially benign, epithelial cells acquire a few distinctly mesenchymal traits that confer to them the ability to invade adjacent tissues and then disseminate to distant tissues. Much of this phenotypic progression towards increased invasiveness depends on the activation of the EMT [5]. Experimental and clinical studies have shown that EMT is an important regulator of ABC protein expression, the active transporters of a broad range of anticancer drugs and the expression of ABC transporters change continuously during EMT [9,10]. We found a correlation between this phenotypic conversion and ABCC4 protein upregulation in HT29 cells overexpressing Snail; thus, in the current study, we propose that ABCC4 protein expression and function are related to epithelial reprogramming in CRC [14]. To support our hypothesis, we first analyzed the *ABCC4* expression levels in CRC tissue. Our analysis of GEO sets showed that *ABCC4* expression was elevated in CRC patient samples compared to normal colon tissue. Further analysis of the same datasets revealed increased expression of mesenchymal markers and decreased expression of E-cadherin in patient samples. We also found a positive correlation between *ABCC4* expression and *TGFβ1/2* receptors and its ligand *TGFβ2*, which were shown to be involved in epithelial conversion induction in cancers. Clinically, ABC transporters were the first and most studied mechanism of resistance associated with MDR. Interestingly, to date, ABCG2 has been recognized as the main drug efflux protein in CRC [21]. Several studies have shown that ABCG2, through its function in xenobiotic clearance, might play an important role in irinotecan resistance. However, other studies comparing the expression of *ABCG2* mRNA in normal colon tissue and tumor tissue showed a decreased expression in tumor tissue. The latter data suggest that primary colon cancer cells exhibit an initial downregulation of *ABCG2* mRNA expression [22]. Our results clearly indicated that ABCC4 is a prevalent drug transporter in tumors in which ABCG2 is downregulated. We showed that HT29 lines overexpressing Snail, which represent a CRC model in the early stages of phenotype conversion, exhibited an upregulated *ABCC4* expression and concomitant downregulated *ABCG2* expression. These results correspond to microarray data (GEO) from patient samples. We found that *ABCC4* was significantly upregulated while *ABCG2* was downregulated in primary tumors compared to normal colon tissues. These data support the hypothesis that CRC may depend on several drug transporters, specifically regulating their expression and executing their function during cancer progression from primary to metastatic disease. Nevertheless, the ABC transporter mRNA expression may have limited reliability with respect to protein function. A very limited number of studies describing the association between the transcriptional and protein overexpression of ABC transporters in cancers have been published [35]. The correlation between the level of ABC proteins and their transporter function in cancers remains to be proven as well. In CRC, the significance of ABCG2 protein measurement in predicting clinical resistance to irinotecan in patients was examined. ABCG2 protein expression analyzed by IHC showed that ABCG2-positive cells were mainly positioned in the front of the carcinomatous tissue (the invasion front), and strong membranous staining was significantly correlated with a higher Dukes’ stage, more lymph nodes, and the presence of distant metastases [36]. However, the role of ABCG2 as a prognostic factor or predictor of irinotecan efficacy in CRC is not well established. The few studies available seem to report discordant results mainly due to the lack of validated assays and standardized reference values for IHC protocols [21,22]. This lack of consistency may also be a result of either cancer heterogeneity and/or an incomplete understanding of the biological role of ABC transporters in cancer progression. In view of the above, the analysis of the mRNA and protein expression levels of specific ABC transporters in relation to their transporter function in various cancer cell subpopulations may have clinical value.

ABCC4 is unique among ABC transporters since its different locations (basolateral/apical membranous versus cytoplasmic) may fundamentally influence its transport function. To date, the mechanism of ABCC4 cellular trafficking has not been elucidated; however, changes in ABCC4 expression led to changes in its localization and function [31,32,33,34]. It is widely accepted that ABC transporters can bind their substrates either from the surroundings of the plasma membrane or intracellular vesicles and transport them out of the cell directly to the external milieu [33,37]. Our results showed a higher level of ABCC4 in the plasma membrane fraction and in EVs from two clones of HT29 cells stably overexpressing Snail, suggesting that the level of ABCC4 expression in CRC determines its functional localization in tumor cells.

To further explore the role of the ABCC4 transporter in CRC progression, we confirmed that ABCC4 upregulation is correlated with the phenotype conversion process in CRC. We measured the ABCC4 protein level in CRC cell lines representing various phenotypes from epithelial to intermediate mesenchymal to strongly mesenchymal. All of these cell lines were either directly derived from primary colorectal cancers of different clinical stages and differentiation grades [25]. We believe that all the lines with clearly defined genetic backgrounds—i.e., methylation and epigenetic status—the occurrence (or not) of *KRAS*, *BRAF*, *SMAD4*, and other mutations were the best experimental models to identify the particular ABCC4 expression status in CRC to mimic a patient-specific approach. ABCC4 expression analysis in those lines showed that cells with an epithelial phenotype (CCD841CoN) expressed less ABCC4 than cells with an intermediate or a strongly mesenchymal phenotype (CaCo-2 and Colo-320, respectively). The highest expression of ABCC4 was observed in Colo-320 cells, strongly mesenchymal cells with the strongest expression of the EMT signature and the highest propensity to local invasion in the analyzed group of cells. These results confirmed that the ABCC4 expression is related to phenotypic transition in CRC.

In addition to xenobiotic efflux, ABCC4 was shown to control the export of endogenous signals, such as cyclic nucleotides and prostaglandins, and their cellular concentration; therefore, ABCC4 plays an important role in other processes. Platelet aggregation, retinal neovascularization, CFTR-mediated secretory diarrhea and fibroblast migration are partially related to ABCC4 transport activity [37]. Among the endogenous substrates of ABCC4, cAMP and cGMP play important roles in the signaling pathways at various stages of the cell migration process, either directly or by activating their corresponding kinases. As ABCC4 was shown to have a higher affinity for cAMP, we tried to monitor the effect of ABCC4 inhibition on the intracellular cAMP levels to further evaluate the significance of ABCC4 protein in CRC progression. We noted that treatment with MK571 increased the intracellular level of cAMP in HT29 cells. However, the effect was more pronounced in HT29 cells that mesenchymal characteristics acquired by Snail overexpression.

The cAMP-PKA pathway is the most relevant mechanism to the outcome of ABCC4 expression. Localized increases in cAMP concentration and cAMP-dependent PKA at the leading edge both play pivotal roles in ensuring the polarity of migrating cells [28,29]. The polarized activation of the cAMP-dependent kinase PKA is not only an essential early step for directional cell migration but is also involved in actin polymerization and cytoskeleton dynamics regulation. We observed that inhibition of ABCC4 increased the intracellular level of cAMP and modulated PKA activity and phospho-serine/tyrosine levels in HT29 cells overexpressing Snail. The diversity of PKA substrates permits the regulation of multiple signaling events based on the subcellular localization of PKA [38]. Studies have shown that at the leading edge, PKA activates small GTPases, such as Rac and Cdc42, which are important for lamellipodia and filopodia formation, respectively, during cell migration [39]. Phosphorylation-activated Rac induces the WASP/WAVE-mediated activation of Apr 2/3 and promotes the formation of dendritic actin network-containing lamellipodia [40]. Additionally, the PKA-dependent phosphorylation of VASP can regulate actin polymerization and hence can control protrusion formation during cell migration. [41].

This observation prompted us to validate the effect of ABCC4 inhibition on cell motility. The obtained results confirmed that HT29 cells with acquired mesenchymal characteristics (by Snail overexpression) are more prone to ABCC4 inhibition, which leads to an increase in the migratory and invasive properties of cells. The same effect—i.e., an increase in migration—was shown in the CaCo-2 line, classified as an intermediate mesenchymal phenotype. Thus, our results showed that in CRC, similar to what was observed in fibroblasts, ABCC4 can regulate cell migration in a cyclic nucleotide-dependent manner. However, the intracellular cyclic nucleotide level is controlled not only by the process involving active efflux transport from the cell but also by phosphodiesterase-mediated hydrolysis. In fibroblasts, the inhibition of ABCC4 function through MK571 treatment or gene knockout showed that the intracellular cAMP level was moderately regulated by ABCC4 near the leading edge of the cells, whereas forskolin and PDE inhibitors strongly elevated the cAMP level inside the cells. Therefore, ABCC4 regulates fibroblast migration through the spatial resolution of cAMP signaling and localized PKA activation at the cell front [18]. Similarly, ABCC4 was shown to modulate the compartmentalization of cAMP signaling in a colon adenocarcinoma cell lines (HT29 and T84), and ABCC4 inhibition with MK571 leads to the accumulation of cAMP at or near the plasma membrane. Interestingly, irinotecan, a first- and second-line chemotherapeutic drug for CRC, was also demonstrated to increase the cAMP levels at or near the plasma membrane to levels comparable to the effect of MK571 in mouse intestinal epithelial cells and human CRC cells [33]. This observation raises the question of whether irinotecan increases the migration rate in CRC cells with a specific phenotype and ABCC4 expression level. Since irinotecan has been reported to be a potential substrate for ABCC4, we reasoned that these drugs, through substrate competition with cAMP, may also elevate intracellular levels of cAMP and increase cell migration and ultimately cancer dissemination. Our results confirmed the above assumption: irinotecan increased CRC migration, and the effect was more pronounced in cells with mesenchymal characteristics.

Taken together, our results indicated that the pharmacological inhibition of ABCC4 regulates cAMP signaling and PKA activity and increases the migratory rate and invasive protrusion formation in CRC. Thus, during the use of ABCC4 inhibitors to reduce chemotherapy resistance or drugs that are potential substrates of ABCC4, the indirect effect on cancer metastasis should be taken into consideration and may be important in selecting a therapy scheme for individual patients. However, the involvement of ABCC4 protein in cell migration is ambiguous. The siRNA silencing of ABCC4 in human retinal microvascular endothelial cells (HRECs) enhanced their migration [42], while pharmacological inhibition of ABCC4 activity or downregulation through RNAi in dendritic cells (DCs) resulted in the reduced migration of DCs [43]. The ambiguous role of cyclic nucleotides in cell migration should also be considered. This observation suggests that various intracellular mechanisms may be responsible for ABCC4 involvement in migration and that the ABCC4 function may be cell type-dependent.

As tumor heterogeneity is accepted and heterogeneity seems particularly pronounced in CRC, patient-derived material analysis is required to further increase the translatability of our findings. The single-cell sequencing of normal tissues, primary tumors, circulating tumor cells, and metastases, combined with cellular analyses and functional validations, will reveal the role of ABCC4 protein in the diverse responses of CRC patients to therapy.

## 4. Materials and Methods

### 4.1. Patients Samples Analysis.

Microarray profiles and datasets of primary CRC were acquired from the public Gene Expression Omnibus (GEO) databases—National Center for Biotechnology Information (NCBI), U.S. National Library of Medicine 8600 Rockville Pike, Bethesda MD, 20894 USA [20] with the following entries: GSE18105, GSE44861, and GSE32323 (Affymetrix Human Genome U133 Plus 2.0 platform). [20] GSE18105 consisted of 110 samples, GSE44861 consisted of 111 samples, and GSE32323 consisted of 34 samples. For each dataset, samples were divided into two defined groups: colon cancer samples (c) and normal colon samples (n)—and the number of analyzed samples in each group was as follows: GSE18105: n_c_ = 94, n_n_ = 16; GSE44861: n_c_ = 56, n_n_ = 55; GSE32323 n_c_ = 17 n_n_ = 18. Next, the obtained data were analyzed using the same microarray ID for each mRNA in every dataset (e.g., 203196_at for ABCC4 analysis). Data were presented as box charts, with the median and all the data points depicted. Statistical analysis was performed using BioVinci version 1.1.5 developed by BioTuring Inc., San Diego, CA, USA, [44]

### 4.2. Cell Culture

Colon cancer cell lines and dermal microvascular endothelium were obtained from American Type Culture Collection (Manassas, VA, USA) and cultured: HT29 and HT29/Snail in McCoy’s 5A medium (LifeTechnologies, Waltham, MA, USA), COLO-320 in RPMI-1640, CCD 841 CoN and CaCo-2 in Eagle’s Minimum Essential Medium (EMEM), HMEC-1 in MCBD-131 medium (Life Technologies, Waltham, MA, USA), all supplemented with 10% FBS (LifeTechnologies, Waltham, MA, USA) and antibiotics—streptomycin and penicillin (Sigma-Aldrich, St. Louis, MO, USA), primocin (Invivogen, San Diego, CA, USA) in a 90–95% humidified atmosphere of 5% CO2. HMEC-1 were additionally supplemented with EGF, hydrocortisone, and L-Glutamine (Sigma-Aldrich, St. Louis, MO, USA). The cells were periodically tested for mycoplasma every 4 weeks using the PlasmoTest (Invivogen, San Diego, CA, USA).

### 4.3. Western Immunoblotting

Proteins isolated from HT-29 cells were extracted with NP-40 lysis buffer (50 mM Tris, pH 8.0, containing 1% Nonidet-Igepal, 150 mM NaCl, 5 mM EDTA) with the Halt protease inhibitor cocktail (Thermo Scientific, Waltham, MA, USA), and the soluble protein fraction was collected through centrifugation. The protein concentrations in the cell lysates were measured with the BCA method (Pierce/Thermo Scientific, Waltham, MA, USA) and were equalized between samples. The protein extracts were subjected to SDS-PAGE analysis and were electro transferred onto PVDF or nitrocellulose membranes (BioRad, Hercules, CA, USA) followed by immunodetection goat anti human ABCC4 #PA5-18315 (Thermo Fisher Scientific), rabbit anty human ABCG2 #ORB155559 (Biorbyt). The control-mouse rabbit anti-α-tubulin antibody conjugated with HRP (NB100-690H) was obtained from Novus Biologicals (Centennial, CO, USA) and used as a loading control. Detection was performed using secondary HRP-conjugated antibodies (Santa Cruz Biotechnology, Dallas, TX, USA) followed by incubation with an enhanced chemiluminescence kit (Thermo Scientific, Waltham, MA, USA) and development with Kodak BioMax Light Film (Eastman Kodak, Rochester, NY, USA).

### 4.4. Biotinylation of Cell-Surface Proteins

HT29 cells were seeded on 75 cm^2^ bottles. After reaching 80% confluence, they were washed 3× with PBS pH 8.0. Next, 2.5 mL freshly made of 2mM biotin (EZ-Link Sulfo -NHS-Biotin Thermo Scientific, Waltham, MA, USA) solution in pH 8.0 PBS was added for 2 h 4 °C. Next, biotin solution was aspirate and cells were washed 3× with cold TBS pH 7.4 solution, then cells were lysed for 30 min using M-PER™ Mammalian Protein Extraction Reagent #78501 (Thermo Scientific, Waltham, MA, USA), centrifuged (20 min, 4 °C) and supernatant were collected and diluted to 1 mg/mL of total proteins. To 1ml of sample, 100 µL of streptavidin agarose slurry was added and incubated overnight at 4 °C on a rocky platform. Agarose/sample was centrifuged and the pellet was washed 4x with lysis solution. Finally, 80 µL of Laemmli buffer supplemented with 2 βME was added and incubated for 10 min at 95 °C, and next the samples were analyzed by SDS-PAGE and Western blot using Ab anti ABCC4 

### 4.5. cAMP Level Measurement

A cAMP level analysis was performed using the cAMP competitive Kit (#581001 Cayman Chemical, Ann Arbor MA, USA). Cells incubated for 24 h w/wo 20 µM MK571 were treated with 0.1M HCl for 20min in RT and assayed according to the manufacturer’s protocol. Calculation was performed using data sheet provided by the Cayman. The cAMP concentration of HT29 control cells was set as 100%, and next all the obtained data were recalculated as the % of control.

### 4.6. Wound Healing (Scratch) Assay

Cells were seeded on 6-well plate or 24-well pate and were grown to confluence; with a 20 μL pipette tip and rinsed twice with PBS. New medium w/wo tested chemical compound was added. The wounded area was visualized after every 2 h using Nikon Eclipse TE 2000-U microscope (Nikon, Japan) or Spark^®^ multimode microplate reader (TECAN, Switzerland)). Wound area was calculated by ImageJ software. Cell motility was estimated through the quantification of the % of recovery using the equation: R (%) = [1 − (wound area at Tt/wound area at T0)] × 100,where T0 is the wounded area at 0 h and Tt is the wounded area after th.

### 4.7. Fluorescent Dequenching (DQ) Gelatine Assay

The surface of 24-well plates was coated with 250 µL 0.1 mg/mL DQ gelatine (Life Technologies, Waltham, MA, USA) overnight at 4 °C and then washed 3× with PBS. Then, 1 × 10^5^ cells/well were added for 24 h to earlier prepared DQ gelatine-coated dishes in full medium supplemented w/wo 20 µM MK571. FITC fluorescence generated by the cleavage of DQ gelatine was measured using a Thermo Labsystem Fluoroscan Ascent reader (ThermoFisher Scientific, Waltham, MA, USA) fitted with FITC excitation and emission filters. Data are presented as the percent of increase above background fluorescence (100%) observed in the control HT-29. 

### 4.8. Trans-Well Invasion and Trans-Well Migration Assays

HT-29 control or HT-29/Snail cells were treated with 20 µM of MK571 for 24 h. Then, cells were trypsinized, washed twice with medium, and transferred (2.5 × 10^4^ cells/chamber) to the upper chamber of Nunc™ Cell Culture Inserts (transwell) 8.0 μm pore diameter (#141006) covered with BD Matrigel (2 h, 0.6 mg/mL of Matrigel—75 µL) for 6 h in 0.1% BSA medium—supplemented w/wo MK571. Full medium in lower chamber was used as chemoattractant. Next, the medium and the Matrigel from the top surface of the membrane was removed, invaded cells on the bottom surface of the membrane were washed 2× with PBS, then fixed for 5 min with 96% ethanol at 4 °C. Cells were dyed at RT as follows: 6 min—hematoxylin, 1 min—1% eosin. Finally, membranes were cut out from chambers, placed on microscope glass and number of cells that migrate into the membrane was counted. CaCo2 cells incubated for 24 h with (MK571 20 µM) or untreated once were seeded on un-coated trans-well inserts (8 µM pores) in the upper chamber in 2% BSA medium (w/wo 20 µM MK571). Full medium in the lower chamber served as chemoattractant for cell migration. Cells were calculated in randomly assigned areas after 3 h of incubation followed by hematoxylin/eosin staining. Minimal and maximal cell counts are shown as the lower and upper extremes by respective whiskers.

### 4.9. PKA Phosphorylation Assay

Cells were seeded on a 6-well plate (5 × 10^5^/well). After 24 h, full growth medium was changed into starving (FBS free) medium for 24 h. Next, 20 µM of MK571 was added to cells for 60, 30, 5, and 1 min. Cells without starving procedure were used as positive control, and cells not treated with MK571 were used as negative control. After washing with PBS, cells were lysed, and SDS/PAGE and Western blot were performed using phospho-(ser/thr) PKA Substrate Antibody #9621 (Cell Signalling, Technology, Danvers, MA, USA). 

### 4.10. WST-1 Cell Viability and Proliferation Assay

A total of 2 × 10^4^ cells per well were seeded on 96-well plate and left for 24 h. Next, 100 µL of fresh medium was added containing irinotecan to a final concentration of 0, 5, 25, 50, 100 µM. Next, after 48 h of incubation 10 µL of WST-1 reagent (ScienCell, Research Lab., Carlsbad, CA, USA) freshly made, #8038 was added for 2 h. Calculation of cell viability was done by OD_450nm_- OD_630nm_ using the Spark multimode microplate reader.

### 4.11. Statistical Analysis

All the experiments were repeated at least three times and the results were expressed as mean ± standard deviation (SD). Statistical evaluation was performed using normality test (Shapiro–Wilk) followed by T-Student test (for normally distributed data) or Mann-Whitney U test (for not normally distributed data). Calculation and graphs were performed using BioVinci version 1.1.5 developed by BioTuring Inc., San Diego, CA, USA, [44]. *p* values < 0.05 were considered statistically significant for all analyses: * *p* < 0.05; ** *p* < 0.005; *** *p* < 0.001, NS—not statistically significant. Pearson’s linear correlation and Spearman correlation (Supplementary Materials) analysis were performed to analyze the correlation between TGFβ1/2 and TGFβR1/2 in CRC tissues with Pearson correlation coefficient (PCC): 0–0.25 no PCC, 0.25–0.5 low PCC, 0.5–0.75 moderately PCC, 0.75–1 strong PCC. Data density distribution is presented in Supplementary Materials, Figure S2 was produced using SinaPlot server [45]. 

The densitometry analysis of WB were performed with *n* = 3 (ABCC4/ABCG2 protein, PKA substrates) or *n* = 2 (EV’s analysis) biological replicates. All the functional tests were performed in triplicate with *n* = 3 of biological replicates.

## 5. Conclusions

During the use of ABCC4 inhibitors to reduce chemotherapy resistance or drugs that are potential substrates of ABCC4, the indirect effect on cancer metastasis should be taken into consideration and may be important in selecting a therapy scheme for individual patients.

## Supplementary Materials

The following are available online at https://www.mdpi.com/2072-6694/12/12/3547/s1: Figure S1 (**A**) Comparison of Pearson’s and Spearman’s correlation values (**B**) Relative densitometry quantification of ABCC4/ABCG2 protein expression level in HT29. (**C**) Relative densitometry quantification of ABCC4 protein expression level in various CRC cells lines. (**D**) Relative densitometry quantification of ABCC4 abundance in HT29 cells EVs. (**E**) Representative films of phosphorylation profile of PKA substrates after ABCC4 inhibition. (**F**) Irinotecan cytotoxicity (IC25 and IC50) for HT29/Snail and control HT29 cells. (**G**) representative images of wound healing of HT-29 control. Figure S2. Data density distribution produced with SinaPlot tools. Figure S3. Original Western Blots.

## References

[1] Henry J.T., Johnson B. (2019). Current and evolving biomarkers for precision oncology in the management of metastatic colorectal cancer. Chin. Clin. Oncol..

[2] Bray F., Ferlay J., Soerjomataram I., Siegel R.L., Torre L.A., Jemal A. (2018). Global cancer statistics 2018: GLOBOCAN estimates of incidence and mortality worldwide for 36 cancers in 185 countries. CA Cancer J. Clin..

[3] Souza e Silva V., Chinen L.T.D., Abdallah E.A., Damascena A., Paludo J., Chojniak R., Dettino A.L.A., de Mello C.A.L., Alves V.S., Fanelli M.F. (2016). Early detection of poor outcome in patients with metastatic colorectal cancer: Tumor kinetics evaluated by circulating tumor cells. Onco Targets Ther..

[4] Jolly M.K., Somarelli J.A., Sheth M., Biddle A., Tripathi S.C., Armstrong A.J., Hanash S.M., Bapat S.A., Rangarajan A., Levine H. (2019). Hybrid epithelial/mesenchymal phenotypes promote metastasis and therapy resistance across carcinomas. Pharmacol. Ther..

[5] Pastushenko I., Blanpain C. (2019). EMT Transition States during Tumor Progression and Metastasis. Trends Cell Biol..

[6] Gupta P.B., Pastushenko I., Skibinski A., Blanpain C., Kuperwasser C. (2019). Phenotypic Plasticity: Driver of Cancer Initiation, Progression, and Therapy Resistance. Cell Stem Cell.

[7] Vu T., Datta P.K. (2017). Regulation of EMT in Colorectal Cancer: A Culprit in Metastasis. Cancers.

[8] El-Awady R., Saleh E., Hashim A., Soliman N., Dallah A., Elrasheed A., Elakraa G. (2016). The Role of Eukaryotic and Prokaryotic ABC Transporter Family in Failure of Chemotherapy. Front. Pharmacol..

[9] Wilkens S. (2015). Structure and mechanism of ABC transporters. F1000Prime Rep..

[10] Jiang Z.S., Sun Y.Z., Wang S.M., Ruan J.S. (2017). Epithelial-mesenchymal transition: Potential regulator of ABC transporters in tumor progression. J. Cancer.

[11] Santamaria P.G., Moreno-Bueno G., Cano A. (2019). Contribution of Epithelial Plasticity to Therapy Resistance. J. Clin. Med..

[12] Saxena M., Stephens M.A., Pathak H., Rangarajan A. (2011). Transcription factors that mediate epithelial–mesenchymal transition lead to multidrug resistance by upregulating ABC transporters. Cell Death Dis..

[13] Kryczka J., Boncela J. (2018). Cell Migration Related to MDR-Another Impediment to Effective Chemotherapy?. Molecules.

[14] Przygodzka P., Papiewska-Pajak I., Bogusz H., Kryczka J., Sobierajska K., Kowalska M.A., Boncela J. (2016). Neuromedin U is upregulated by Snail at early stages of EMT in HT29 colon cancer cells. Biochim. Biophys. Acta.

[15] Wen J., Luo J., Huang W., Tang J., Zhou H., Zhang W. (2015). The Pharmacological and Physiological Role of Multidrug-Resistant Protein 4. J. Pharmacol. Exp. Ther..

[16] Li C., Krishnamurthy P.C., Penmatsa H., Marrs K.L., Wang X.Q., Zaccolo M., Jalink K., Li M., Nelson D.J., Schuetz J.D. (2007). Spatiotemporal coupling of cAMP transporter to CFTR chloride channel function in the gut epithelia. Cell.

[17] Sinha C., Ren A., Arora K., Moon C.S., Yarlagadda S., Zhang W., Cheepala S.B., Schuetz J.D., Naren A.P. (2013). Multi-drug Resistance Protein 4 (MRP4)-mediated Regulation of Fibroblast Cell Migration Reflects a Dichotomous Role of Intracellular Cyclic Nucleotides. J. Biol. Chem..

[18] Sinha C., Ren A., Arora K., Moon C.S., Yarlagadda S., Woodrooffe K., Lin S., Schuetz J.D., Ziady A.G., Naren A.P. (2015). PKA and actin play critical roles as downstream effectors in MRP4-mediated regulation of fibroblast migration. Cell. Signal..

[19] Delou J., Souza A.S.O., Souza L.C.M., Borges H.L. (2019). Highlights in Resistance Mechanism Pathways for Combination Therapy. Cells.

[20] Geo (2020). GEO2R-GEO-NCBI. https://www.ncbi.nlm.nih.gov/geo.

[21] Candeil L., Gourdier I., Peyron D., Vezzio N., Copois V., Bibeau F., Orsetti B., Scheffer G.L., Ychou M., Khan Q.A. (2004). ABCG2 Overexpression in Colon Cancer Cells Resistant to SN38 and in Irinotecan-Treated Metastases. Int. J. Cancer.

[22] Nielsen D.L., Palshof J.A., Brünner N., Stenvang J., Viuff B.M. (2017). Implications of ABCG2 Expression on Irinotecan Treatment of Colorectal Cancer Patients: A Review. Int. J. Mol. Sci..

[23] Schlicker A., Beran G., Chresta C.M., McWalter G., Pritchard A., Weston S., Runswick S., Davenport S., Heathcote K., Castro D.A. (2012). Subtypes of primary colorectal tumors correlate with response to targeted treatment in colorectal cell lines. BMC Med. Genom..

[24] Melo F.D.S.E., Wang X., Jansen M., Fessler E., Trinh A., Rooij L.P.M.H.d., Jong J.H.d., Boer O.J.d., Leersum R.v., Bijlsma M.F. (2013). Poor-prognosis colon cancer is defined by a molecularly distinct subtype and develops from serrated precursor lesions. Nat. Med..

[25] Christensen J., El-Gebali S., Natoli M., Sengstag T., Delorenzi M., Bentz S., Bouzourene H., Rumbo M., Felsani A., Siissalo S. (2012). Defining new criteria for selection of cell-based intestinal models using publicly available databases. BMC Genom..

[26] Maacha S., Bhat A.A., Jimenez L., Raza A., Haris M., Uddin S., Grivel J.-C. (2019). Extracellular vesicles-mediated intercellular communication: Roles in the tumor microenvironment and anti-cancer drug resistance. Mol. Cancer.

[27] Przygodzka P., Papiewska-Pająk I., Bogusz-Koziarska H., Sochacka E., Boncela J., Kowalska M.A. (2019). Regulation of miRNAs by Snail during epithelial-to-mesenchymal transition in HT29 colon cancer cells. Sci. Rep..

[28] Kryczka J., Papiewska-Pajak I., Kowalska M.A., Boncela J. (2019). Cathepsin B Is Upregulated and Mediates ECM Degradation in Colon Adenocarcinoma HT29 Cells Overexpressing Snail. Cells.

[29] O‘Brien E.D., Krapf D., Cabada M.O., Visconti P.E., Arranz S.E. (2011). Transmembrane adenylyl cyclase regulates amphibian sperm motility through protein kinase A activation. Dev. Biol..

[30] Liang C.C., Park A.Y., Guan J.L. (2007). In vitro scratch assay: A convenient and inexpensive method for analysis of cell migration in vitro. Nat. Protoc..

[31] Kryczka J., Przygodzka P., Bogusz H., Boncela J. (2017). HMEC-1 adopt the mixed amoeboid-mesenchymal migration type during EndMT. Eur. J. Cell Biol..

[32] Schneider C.A., Rasband W.S., Eliceiri K.W. (2012). NIH Image to ImageJ: 25 years of image analysis. Nat. Methods.

[33] Moon C., Zhang W., Ren A., Arora K., Sinha C., Yarlagadda S., Woodrooffe K., Schuetz J.D., Valasani K.R., de Jonge H.R. (2015). Compartmentalized accumulation of cAMP near complexes of multidrug resistance protein 4 (MRP4) and cystic fibrosis transmembrane conductance regulator (CFTR) contributes to drug-induced diarrhea. J. Biol. Chem..

[34] Pavillard V., Agostini C., Richard S., Charasson V., Montaudon D., Robert J. (2002). Determinants of the cytotoxicity of irinotecan in two human colorectal tumor cell lines. Cancer Chemother. Pharmacol..

[35] Jensen N.F., Stenvang J., Beck M.K., Hanakova B., Belling K.C., Do K.N., Viuff B., Nygard S.B., Gupta R., Rasmussen M.H. (2015). Establishment and characterization of models of chemotherapy resistance in colorectal cancer: Towards a predictive signature of chemoresistance. Mol. Oncol..

[36] Giampieri R., Scartozzi M., Loretelli C., Piva F., Mandolesi A., Lezoche G., Del Prete M., Bittoni A., Faloppi L., Bianconi M. (2013). Cancer stem cell gene profile as predictor of relapse in high risk stage II and stage III, radically resected colon cancer patients. PLoS ONE.

[37] Fletcher J.I., Haber M., Henderson M.J., Norris M.D. (2010). ABC transporters in cancer: More than just drug efflux pumps. Nat. Rev. Cancer.

[38] Arora K., Sinha C., Zhang W., Ren A., Moon C.S., Yarlagadda S., Naren A.P. (2013). Compartmentalization of cyclic nucleotide signaling: A question of when, where, and why?. Pflugers Arch..

[39] Krause M., Dent E.W., Bear J.E., Loureiro J.J., Gertler F.B. (2003). Ena/VASP proteins: Regulators of the actin cytoskeleton and cell migration. Annu. Rev. Cell Dev. Biol..

[40] Le Clainche C., Carlier M.F. (2008). Regulation of actin assembly associated with protrusion and adhesion in cell migration. Physiol. Rev..

[41] Hara Y., Sassi Y., Guibert C., Gambaryan N., Dorfmuller P., Eddahibi S., Lompre A.M., Humbert M., Hulot J.S. (2011). Inhibition of MRP4 prevents and reverses pulmonary hypertension in mice. J. Clin. Investig..

[42] Tagami M., Kusuhara S., Imai H., Uemura A., Honda S., Tsukahara Y., Negi A. (2010). MRP4 knockdown enhances migration, suppresses apoptosis, and produces aggregated morphology in human retinal vascular endothelial cells. Biochem. Biophys. Res. Commun..

[43] Schaletzki Y., Kromrey M.L., Broderdorf S., Hammer E., Grube M., Hagen P., Sucic S., Freissmuth M., Volker U., Greinacher A. (2017). Several adaptor proteins promote intracellular localisation of the transporter MRP4/ABCC4 in platelets and haematopoietic cells. Thromb. Haemost..

[44] INCB (2020). BioTuring Browser|BioTuring. www.bioturing.com.

[45] Sidiropoulos N., Sohi S.H., Pedersen T.L., Porse B.T., Winther O., Rapin N., Bagger F.O. (2018). SinaPlot: An Enhanced Chart for Simple and Truthful Representation of Single Observations Over Multiple Classes. J. Comput. Gr. Stat..

