# Supplementary material for: Implications of ABCC4–Mediated cAMP Efflux for CRC Migration
Source: Cancers (Basel). 2020 Nov 27;12(12):3547. doi: 10.3390/cancers12123547 (PMC7760996; doi:10.3390/cancers12123547)
Supplement: Supplementary file 1 [file cancers-12-03547-s001.pdf]

Supplementary Materials: Implications of ABCC4–Mediated cAMP Eflux for CRC Migration

Jakub Kryczka, Ewelina Sochacka, Izabela Papiewska-Pająk and Joanna Boncela

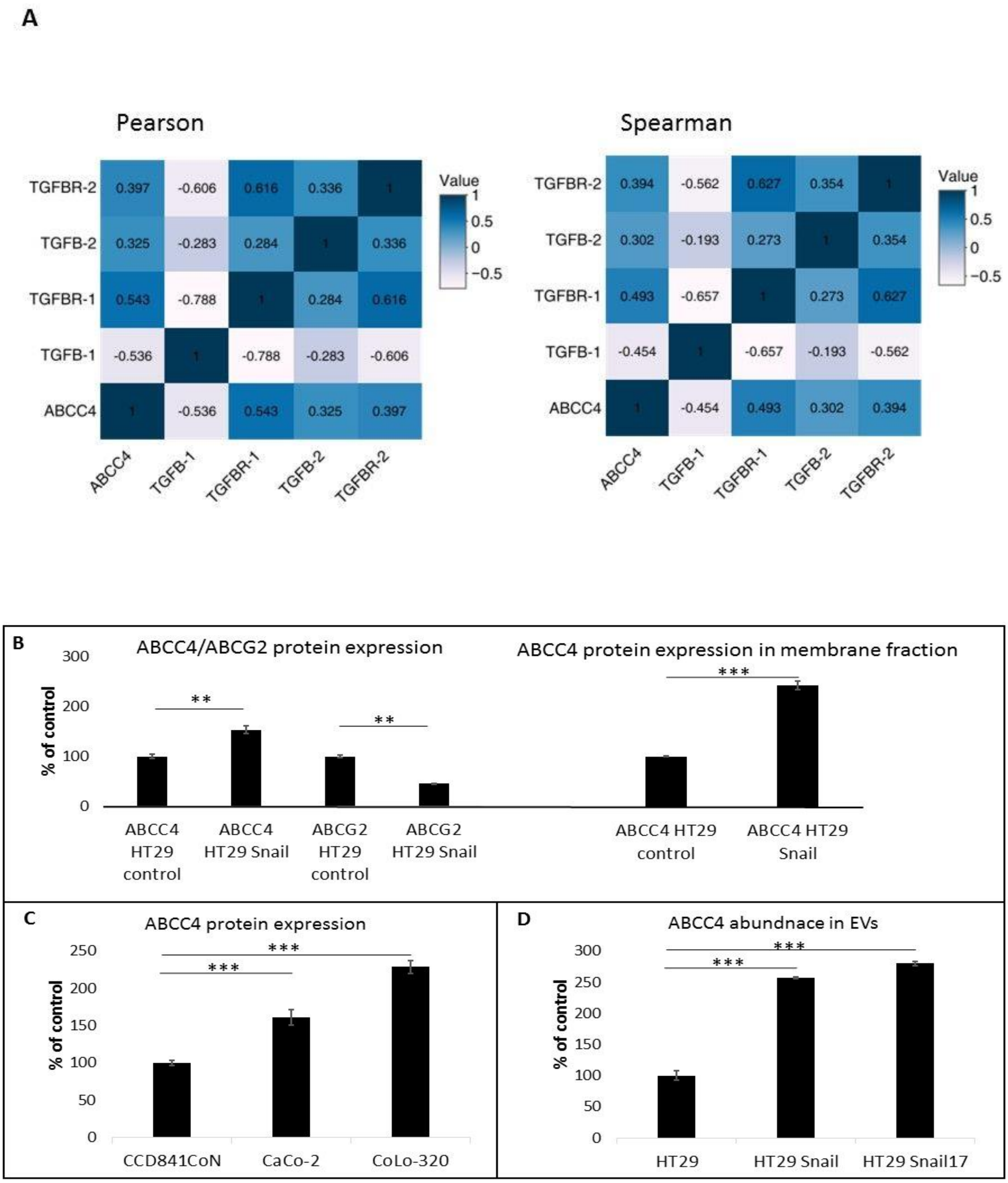

E

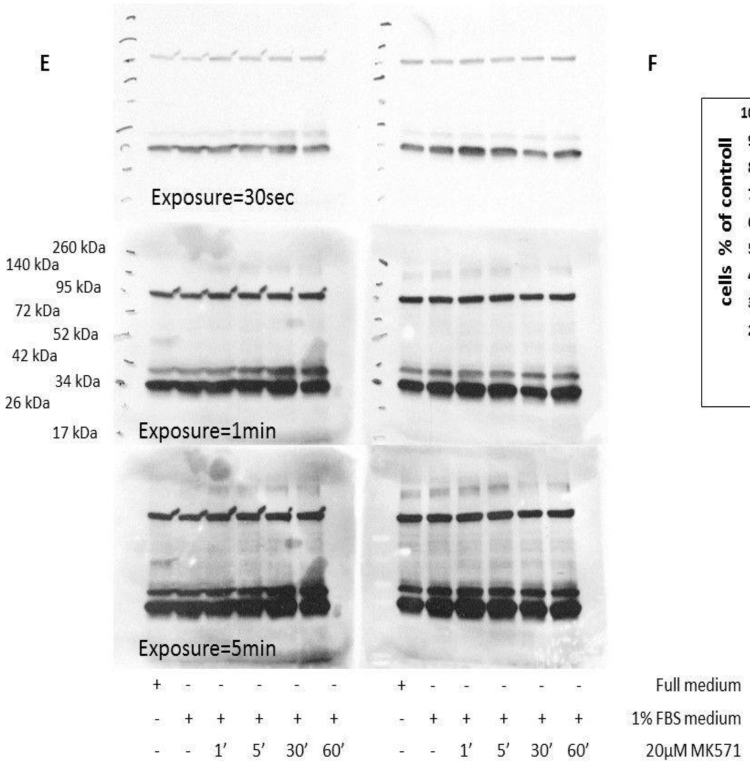

F

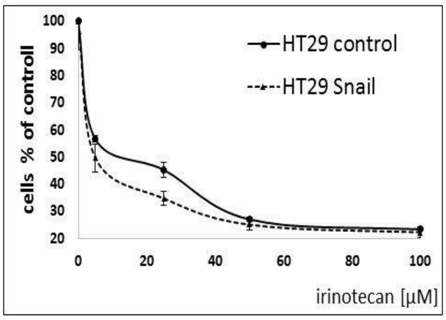

G

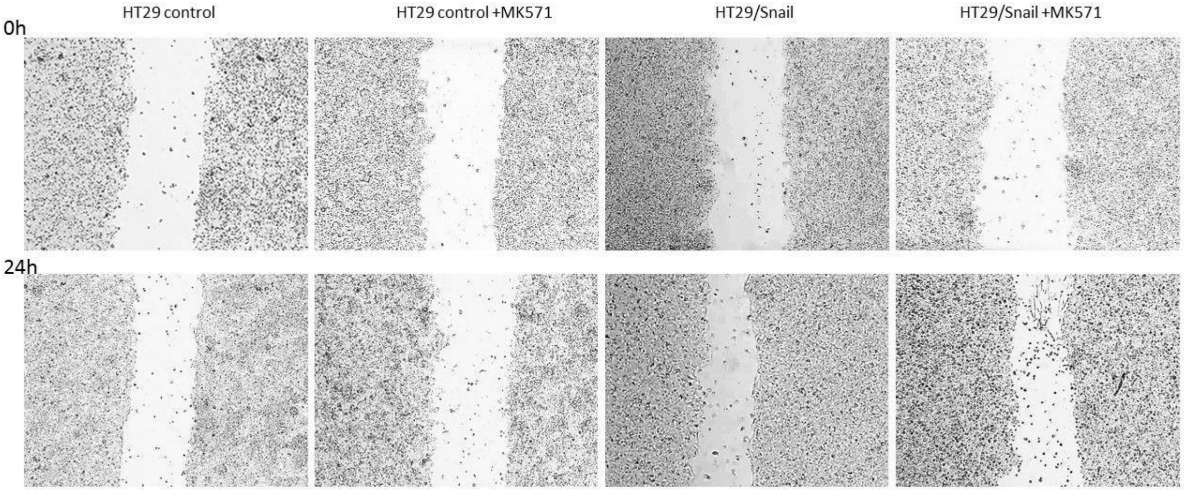

Figure S1. A) Comparison of Pearson's and Spearman's correlation values **B)** Relative densitometry quantification of ABCC4/ABCG2 protein expression level in HT29 cells (left panel). Relative densitometry quantification of ABCC4 protein expression in membrane fraction in HT29 cells (right panel). Expression of respective proteins in HT29 control cells were set as control 100%. **C)** Relative densitometry quantification of ABCC4 protein expression level in various CRC cells lines. CCD841CoN was set as control 100%. **D)** Relative densitometry quantification of ABCC4 abundance in HT29 cells EVs. Expression of ABCC4 in EVs from HT29 control cells were set as control 100%; statistic significance calculated using T-test *P < 0.05; **P < 0.005; ***P < 0.001. **E)** Representative films of phosphorylation profile of PKA substrates after ABCC4 inhibition in HT29/Snail (left panel) and control HT29 cells (right panel) from 3 different exposure time. **F)** Irinotecan cytotoxicity (IC25 and IC50) for HT29/Snail and control HT29 cells. G) representative images of wound healing of HT-29 control w/wo MK571 and HT-29/Snail w/wo MK571.

Figure S2

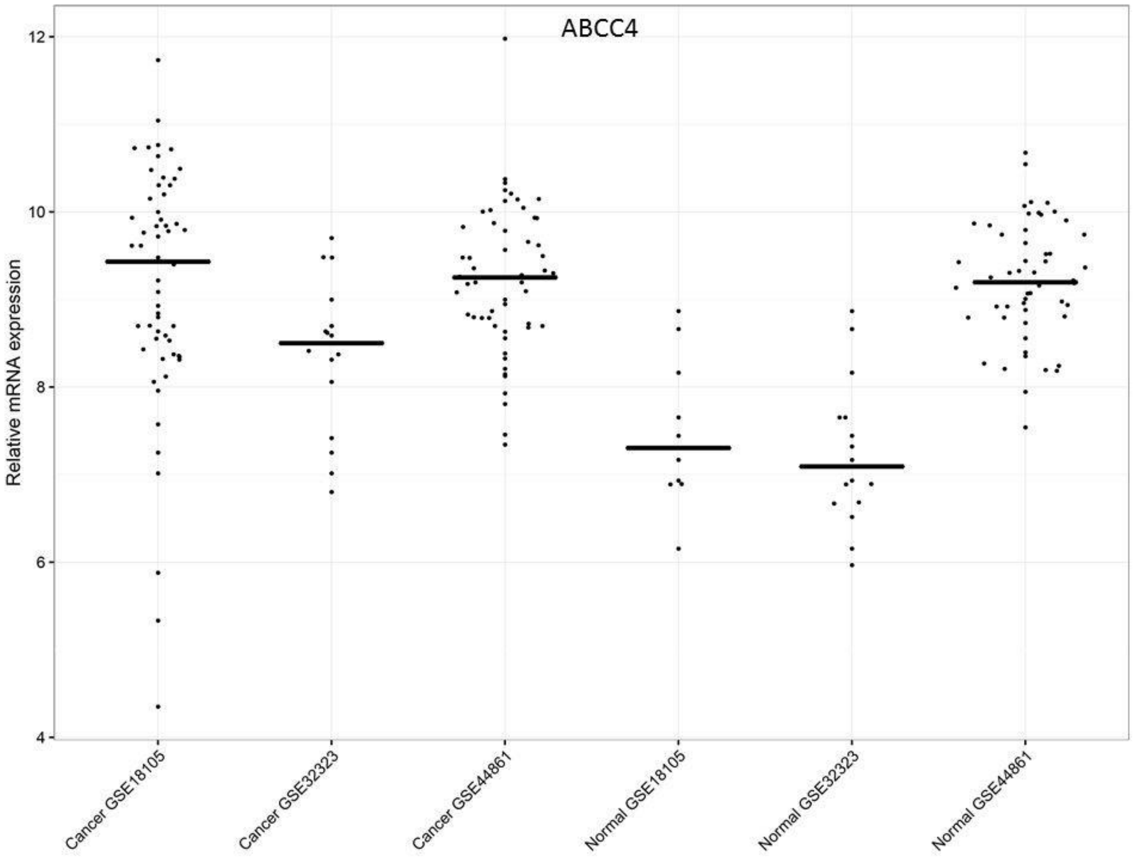
ABCC4
Relative mRNA expression
12
10
8
6
4
Cancer GSE18105
Cancer GSE32323
Cancer GSE44861
Normal GSE18105
Normal GSE32323
Normal GSE44861

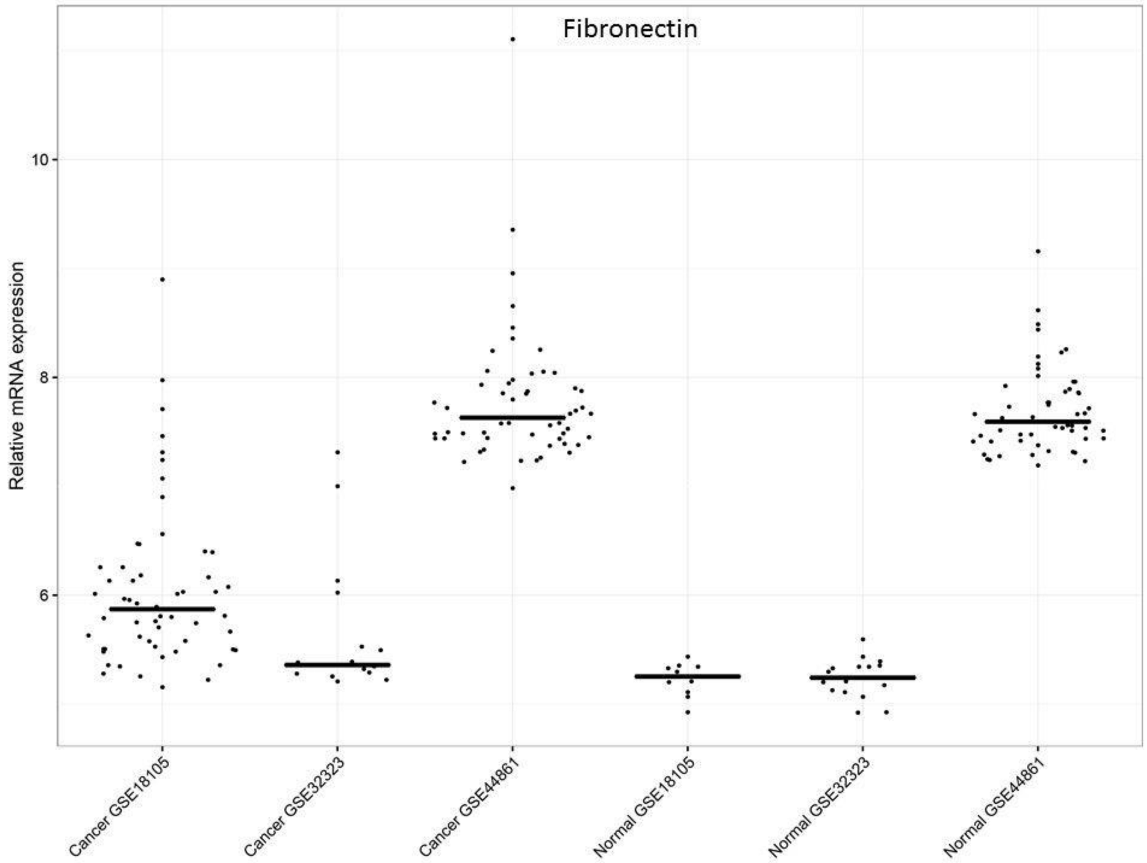
Fibronectin
Relative mRNA expression
10
8
6
Cancer GSE18105
Cancer GSE32323
Cancer GSE44861
Normal GSE18105
Normal GSE32323
Normal GSE44861

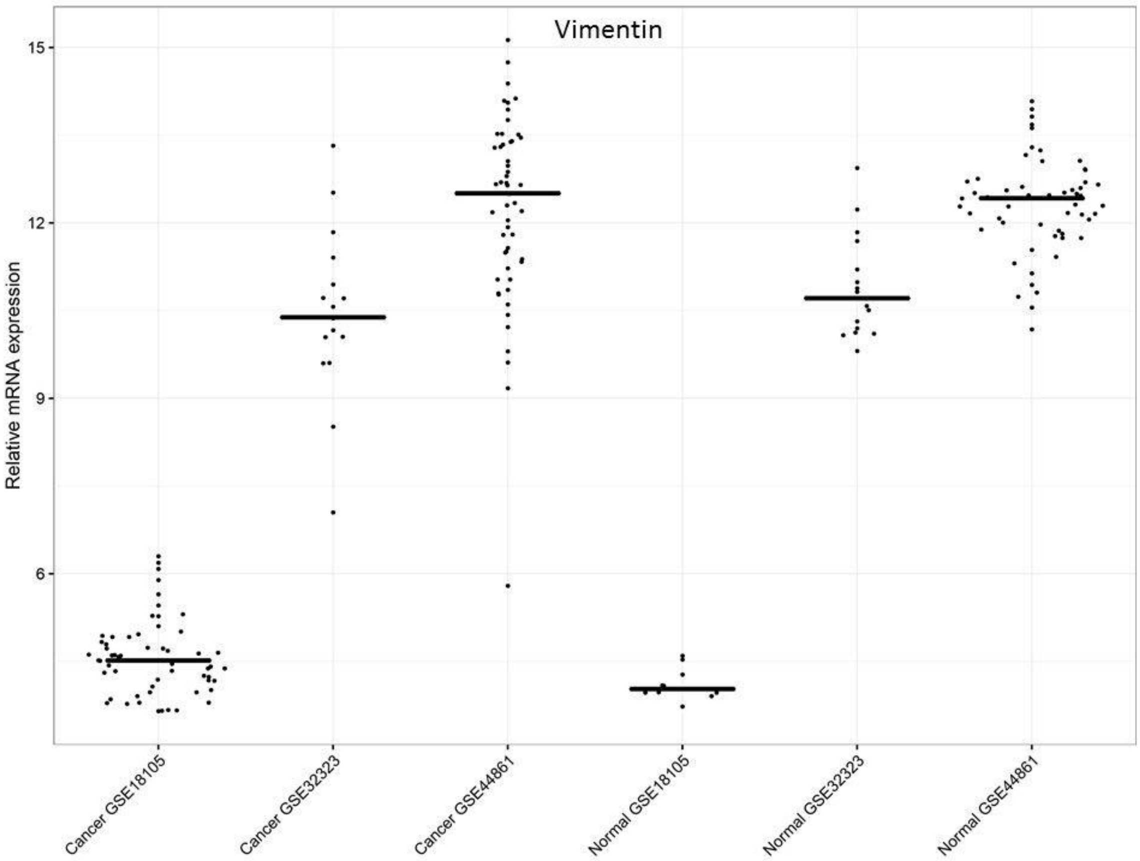
Vimentin
Relative mRNA expression
15
12
9
6
Cancer GSE18105
Cancer GSE32323
Cancer GSE44861
Normal GSE18105
Normal GSE32323
Normal GSE44861

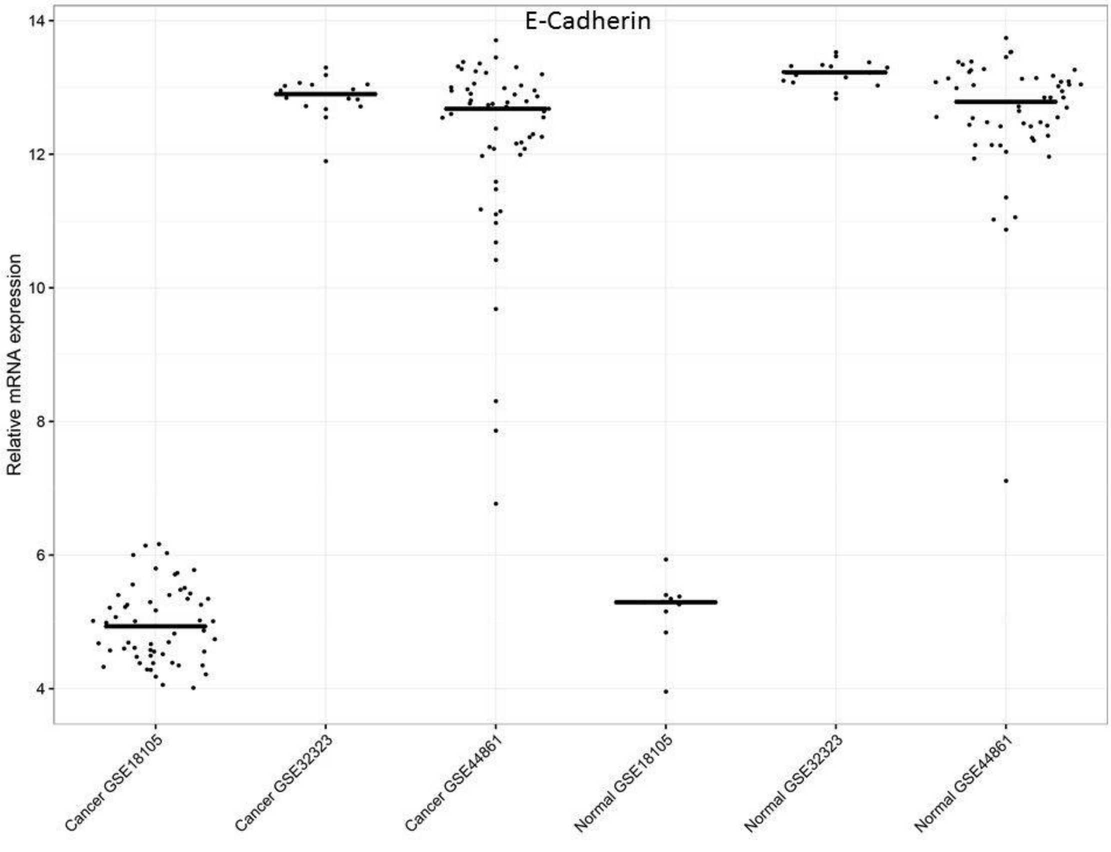
E-Cadherin
Relative mRNA expression
14
12
10
8
6
4
Cancer GSE18105
Cancer GSE32323
Cancer GSE44861
Normal GSE18105
Normal GSE32323
Normal GSE44861

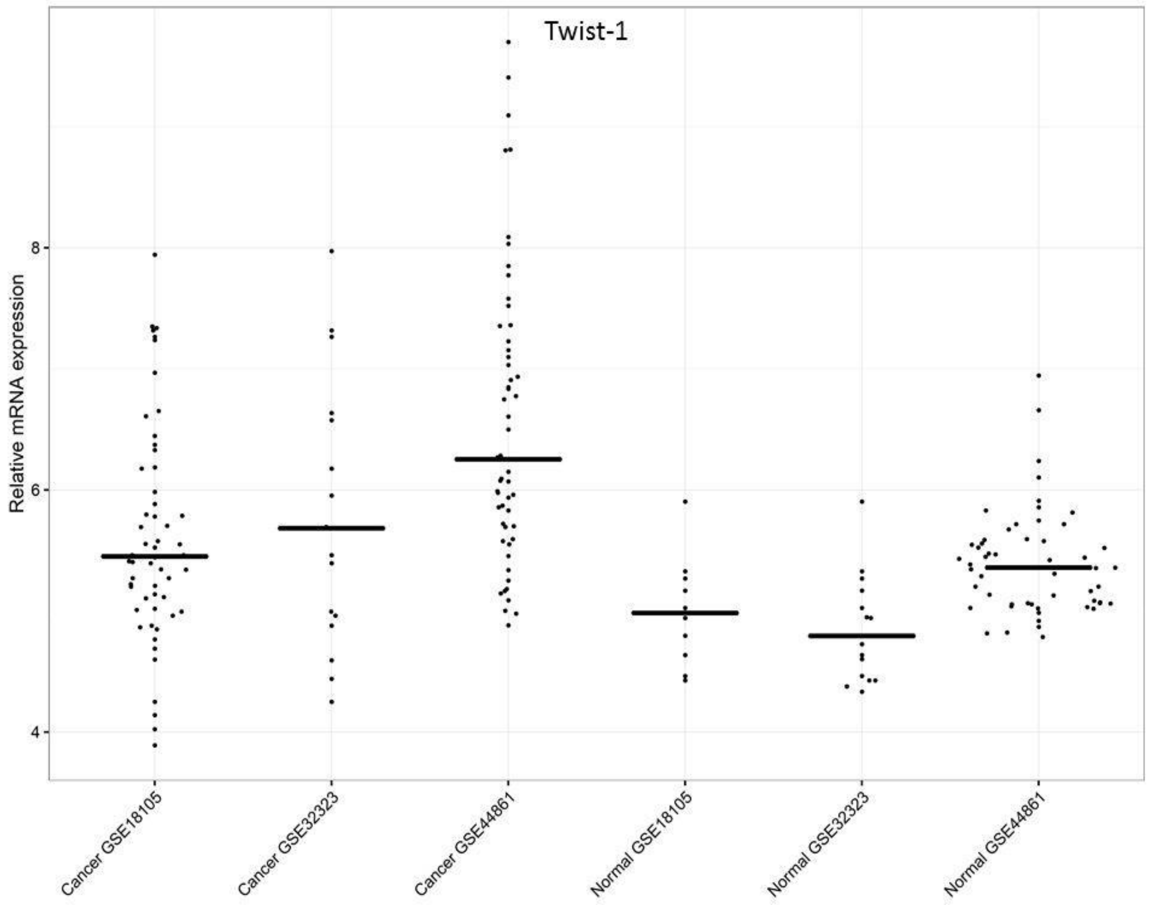
Twist-1
Relative mRNA expression
8
6
4
Cancer GSE18105
Cancer GSE32323
Cancer GSE44861
Normal GSE18105
Normal GSE32323
Normal GSE44861

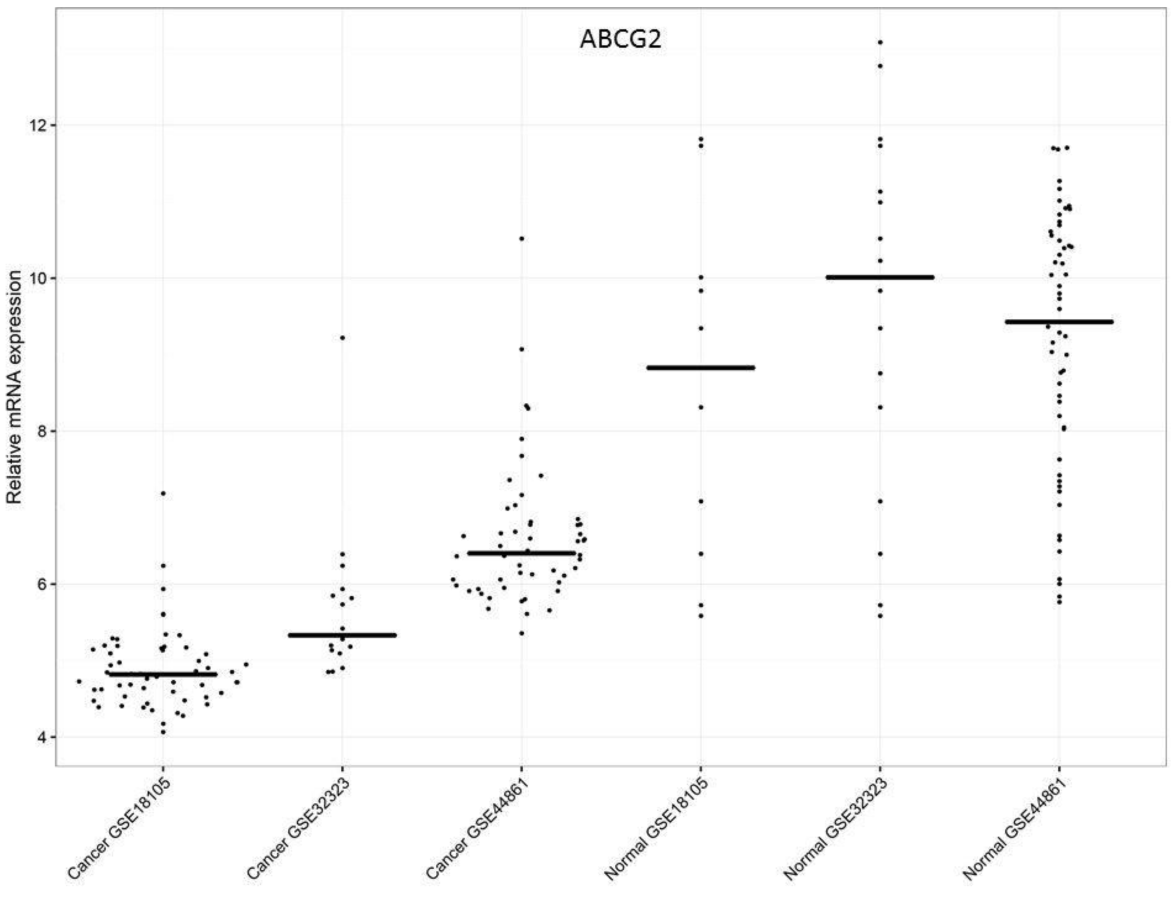
ABCG2
Relative mRNA expression
12
10
8
6
4
Cancer GSE18105
Cancer GSE32323
Cancer GSE44861
Normal GSE18105
Normal GSE32323
Normal GSE44861

Figure S2. Data density distribution produced with SinaPlot tools. (https://www.tandfonline.com/doi/abs/10.1080/10618600.2017.1366914.

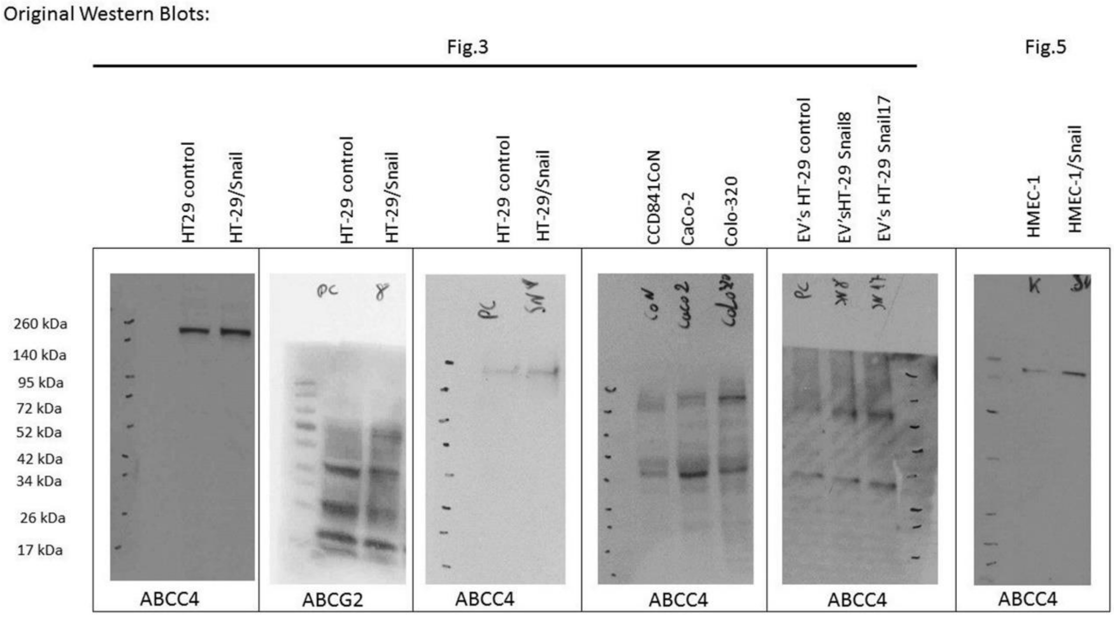

Figure S3. Original Western Blots.
